# Hippocampal expression of murine IL-4 results in exacerbation of amyloid deposition

**DOI:** 10.1186/1750-1326-7-36

**Published:** 2012-07-29

**Authors:** Paramita Chakrabarty, Li Tianbai, Amanda Herring, Carolina Ceballos-Diaz, Pritam Das, Todd E Golde

**Affiliations:** 1 Center for Translational Research in Neurodegenerative Disease, Department of Neuroscience, University of Florida, 1275 Center Drive, Gainesville, PO Box #100159, FL, 32610, USA; 2 Department of Neuroscience, Mayo Clinic College of Medicine, 4500 San Pablo Rd S, Jacksonville, FL, 32224, USA

**Keywords:** Interleukin 4, Inflammation, Adeno-associated virus, Hippocampus, Amyloid plaque, Amyloid precursor protein

## Abstract

**Background:**

Pro-inflammatory stimuli, including cytokines like Interleukin-1β, Interleukin-6 and Interferon-γ, in the brain have been proposed to exacerbate existing Alzheimer’s disease (AD) neuropathology by increasing amyloidogenic processing of APP and promoting further Aβ accumulation in AD. On the other hand, anti-inflammatory cytokines have been suggested to be neuroprotective by reducing neuroinflammation and clearing Aβ. To test this hypothesis, we used adeno-associated virus serotype 1 (AAV2/1) to express an anti-inflammatory cytokine, murine Interleukin-4 (mIL-4), in the hippocampus of APP transgenic TgCRND8 mice with pre-existing plaques.

**Results:**

mIL-4 expression resulted in establishment of an “M2-like” phenotype in the brain and was accompanied by exacerbated Aβ deposition in TgCRND8 mice brains. No change in holo APP or APP C terminal fragment or phosphorylated tau levels were detected in mIL-4 expressing CRND8 cohorts. Biochemical analysis shows increases in both SDS soluble and insoluble Aβ. mIL-4 treatment attenuates soluble Aβ40 uptake by microglia but does not affect aggregated Aβ42 internalization by microglia or soluble Aβ40 internalization by astrocytes.

**Conclusions:**

Short term focal mIL-4 expression in the hippocampus leads to exacerbation of amyloid deposition in vivo, possibly mediated by acute suppression of glial clearance mechanisms. Given that recent preclinical data from independent groups indicate engagement of the innate immune system early on during disease pathogenesis may be beneficial, our present study strongly argues for a cautious re-examination of unwarranted side–effects of anti-inflammatory therapies for neurodegenerative diseases, including AD.

## Background

Amyloid β (Aβ) plaques constitute a hallmark pathological feature of Alzheimer’s disease (AD), the most prevalent neurodegenerative disorder. Neuroinflammation has been hypothesized to play a pathogenic role in the development of sporadic AD, particularly because pro-inflammatory cytokines and chemokines colocalize with neurodegenerative pathology in both AD patient brains as well as in transgenic mouse models of AD type pathology (reviewed in [1]). However, the placement of the inflammatory response in AD neurodegenerative cascade is still debated, with conflicting ideas of innate immune activation either being the trigger, a homeostatic response mechanism or a bystander phenomenon associated with the disease pathology [2].

Anti-inflammatory cytokines, such as Interleukin (IL) -4, lead to the suppression of pro-inflammatory responses in macrophages, microglia, T cells, and astrocytes [3]. Anti-inflammatory cytokines are thought to enhance Aβ degradation through phagocytosis and receptor-mediated uptake leading to the abrogation of Aβ induced cell death in APP transgenic mice [4,5] and primary glia [6]. Down regulation of IL-4 receptors contributes to aging related cognitive impairment [7]. Consequently, IL-4 or minocycline treatment leads to restored synaptic activity in rats following intracerebroventricular infusion of Aβ [8]. However, emerging evidence has also shown that activation of the innate immune system may constitute a beneficial defense mechanism to clear Aβ from the CNS [9]. Data from our lab [10-12] and others [13-17] have demonstrated that glial activation can effectively clear Aβ plaques. It is generally thought that although a sustained inflammatory response is neurotoxic, activation of the innate immune system can indeed have a beneficial function by clearing debris and possibly promoting repair.

In an effort to further understand the role of neuroinflammation on Aβ plaque pathology and specifically to test the role of anti-inflammatory cytokines on Aβ pathology, we utilized recombinant adeno-associated virus serotype 1 (rAAV2/1) to overexpress murine IL-4 (mIL-4) in the hippocampus of Amyloid precursor protein (APP) transgenic mice with pre-existing amyloid plaques. Our results show that mIL-4 expression resulted in exacerbated Aβ deposition in APP transgenic mice brains after 6 weeks of expression. Biochemical analysis of mIL-4 overexpressing mice brains and phagocytosis assays in primary murine glia suggests that mIL-4 expression leads to increased Aβ levels possibly as a result of reduced glia phagocytosis.

## Results

**Figure 1:**
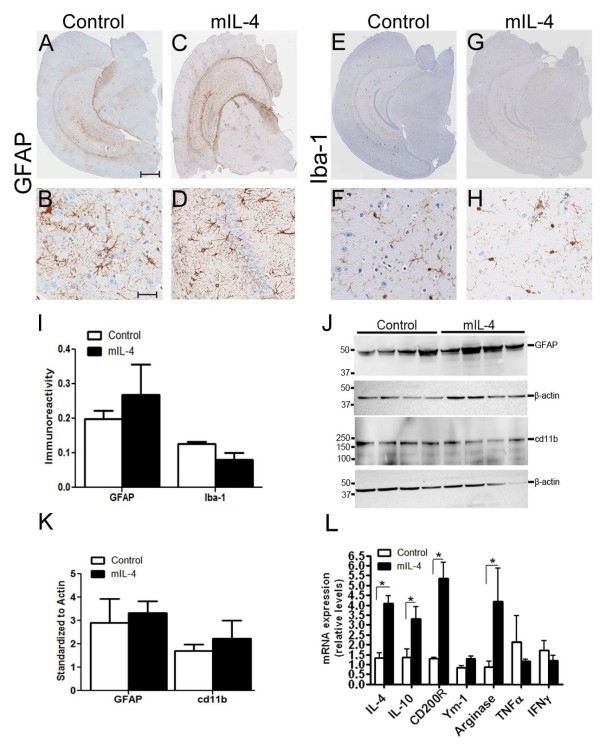
**AAV2/1 mediated expression of mIL-4 in TgCRND8 mice results in M2 phenotype. A-D.** rAAV2/1-mIL-4 or rAAV2/1-EGFP (Control) was injected into the hippocampus of 4 month old TgCRND8 mice and analyzed after 6 weeks. Representative images of GFAP immunoreactivity in paraffin embedded whole brain sections (A, C) and higher magnification of the hippocampus (B, D) is shown. *Scale Bar,* 600 μm (A, C) and 25 μm (B, D). (*n* = 5-6/group). **E-H.** Representative images of Iba-1 immunoreactivity in paraffin embedded sections of 5.5 month old TgCRND8 mice injected with rAAV2/1 mIL-4 or rAAV2/1-EGFP (Control). Whole brain sections (E, G) and the corresponding hippocampus (F, H) are shown. *Scale Bar,* 600 μm (E, G) and 25 μm (F, H). (*n* = 5-6/group). **I**. Densitometric analysis of GFAP and Iba-1 immunostaining is shown. The hippocampal region was selected and Aperio “positive pixel count” program was used to measure percent positivity by averaging intensity of positive staining in the annotated region. (*n* = 4/group; *p* > 0.05, *t* test). Data represents mean ± sem. **J-K.** Representative immunoblot (J) and densitometric analysis of normalized levels of GFAP and cd11b (K) obtained from 5.5 month old TgCRND8 mice injected with rAAV2/1 mIL-4 or rAAV2/1-EGFP (n = 5/group; *p* > 0.05, *t* test). Data represents mean ± sem. **L.** Expression of glial activation markers and cytokines were determined in 5.5 month old mIL-4 expressing TgCRND8 mice compared to EGFP expressing age-matched controls using real time Q-PCR. Data, expressed as relative levels of mRNA expression, represents averaged fold change values obtained from mIL-4 expressing mice, relative to averaged values obtained from EGFP expressing mice. (*n* = 4/group; *p < 0.05, *t* test). Data represents mean ± sem.

To explore the role of anti-inflammatory cytokines in regulating Aβ accumulation in the CNS, we have used recombinant adeno-associated virus serotype 1 (rAAV2/1) to express mIL-4 in the brains of APP transgenic TgCRND8 mice. Recombinant AAV2 plasmids were packaged in AAV serotype 1 capsid as described previously [10] and rAAV2/1 viruses expressing mIL-4 or EGFP under the control of the cytomegalovirus enhancer/chicken β-actin promoter were used for further experiments. Adult TgCRND8 mice were stereotaxically injected with AAV2/1 constructs (1x10^13^ particles/ml) into the CA layer of the hippocampus at 4 months (after Aβ plaque deposition has started) and were analyzed after 6 weeks (*n* = 6 for rAAV1-mIL-4; *n* = 6 for rAAV1-EGFP). Immunohistochemical analysis with anti-EGFP antibody shows that the viral transgene is predominantly expressed in the hippocampal CA neurons, neuronal projections in the cortex and thalamus, and some overlying cortical neurons following 6 week expression of AAV1-EGFP (Additional file 1: Figure S1, A-I). No detectable expression was noted in the midbrain, olfactory bulb or cerebellum. In previous studies AAV2/1-EGFP expression had minimal effects on amyloid pathology or gliosis when compared to naïve uninjected mice [10,18,19]; so, AAV1-EGFP injected animals were used as the control cohort in this study. For adult injected mice, the brain was coronally dissected 1 mm anterior and posterior to the point of injection and used for subsequent analysis. We did not observe any significant changes in GFAP immunoreactive astrocytes (Figure 1, A-D) or Iba-1 reactive microglia (Figure 1, E-H) in the hippocampus of mIL-4 expressing mice compared to control mice. A careful quantification of the histological staining (GFAP and Iba-1) in mouse hippocampus using “Positive Pixel Count” program (Aperio, CA) showed that though mIL-4 expressing mice had less microglial activation in and around the injection area there were no significant changes in either astrocyte or microglial levels overall (Figure 1, I). Immunoblotting analysis with hippocampal lysates also showed essentially unchanged astrogliosis profile in mIL-4 and control cohorts (Figure 1, J-K). Analysis of mRNA from injected mice hippocampus showed significantly increased levels of mIL-4 (3.13 times over control) and mIL-10 (2.4 times over control) (Figure 1, L). No significant change in levels of pro-inflammatory cytokines, TNF-α and IFN-γ, or cd11b (data not shown) was seen. Furthermore, this was accompanied by an increase in Arginase (4 times over control) (Figure 1, L), suggesting an establishment of an alternative “M2a” microglial phenotype [1,20]. Increased levels of CD200R (4.2 times over control) was also noted, similar to previous observations [21].

**Figure 2:**
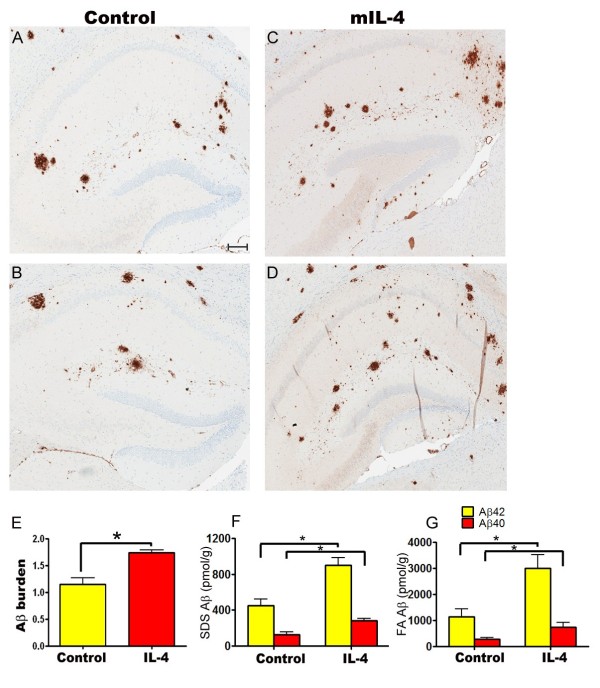
**Increased Aβ deposition in AAV1-mIL-4 expressing TgCRND8 mice. A-D.** 4 month old TgCRND8 mice were stereotaxically injected in the hippocampus with either AAV1-mIL-4 (C, D) or AAV1-EGFP (A, B) and sacrificed after 6 weeks (*n* = 5-6/group). Representative brain sections stained with 33.1.1 antibody (pan Aβ 1–16) depict increased Aβ deposition in mIL-4 expressing mice (C, D) compared to controls (A-B) in the immediate vicinity of the injection site. *Scale Bar,* 150 μm. **E.** Aβ plaque burden analysis shows a significantly increased amyloid deposition in mIL-4 injected mice compared to control EGFP injected mice (*n* = 5/group). (*p < 0.05, *t* test). Data represents mean ± sem. **F-G.** Biochemical analyses of Aβ42 and Aβ40 levels by ELISA show significantly increased SDS (F) and formic acid (G) extractable Aβ levels in mIL-4 injected mice compared to controls (*n* = 5/group). (*p < 0.05, *t* test). Data represents mean ± sem.

**Figure 3:**
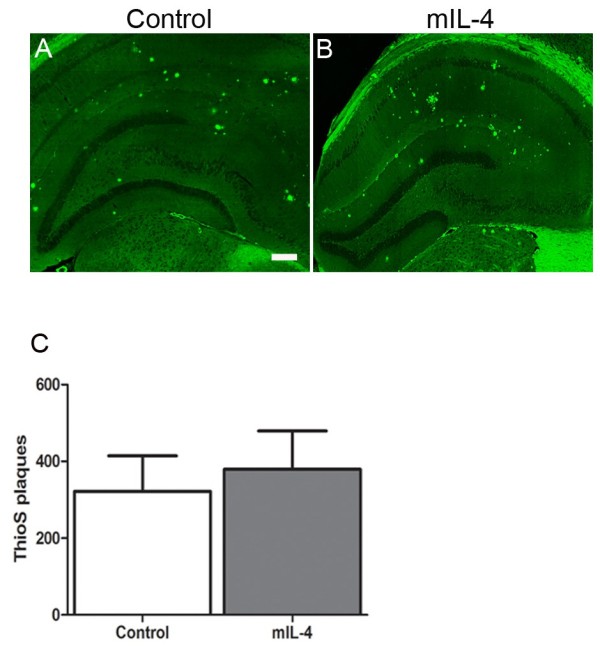
**No significant change in cored plaques seen in AAV1-mIL-4 expressing TgCRND8 mice. A-B.** Representative fluorescent images of Thioflavin S stained hippocampi from 5.5 month old TgCRND8 mice injected with rAAV2/1 mIL-4 (B) or rAAV2/1-EGFP (Control). *Scale Bar,* 200 μm. **C.** A comparison of the total number of Thioflavin S stained “cored” plaques from the forebrain of 5.5 month old TgCRND8 mice injected with rAAV2/1 mIL-4 or rAAV2/1-EGFP is depicted. Data represents mean ± sem. (*n* = 5/group; p = 0.0542, *t* test).

**Figure 4:**
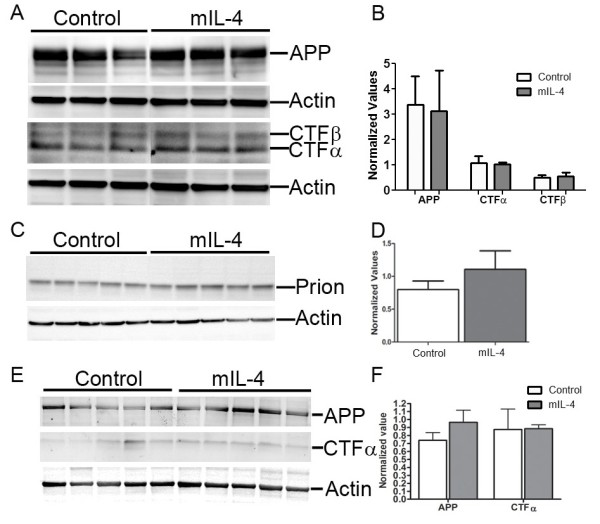
**mIL-4 does not affect human APP transgene expression or CTF production. A-B.** Representative anti CT20 immunoblot showing no significant changes in APP, CTFα and CTFβ levels in AAV1-mIL-4 expressing TgCRND8 compared to age-matched controls (A). Intensity analysis (mean ± sem) of anti CT20 immunoreactive APP and CTFα levels was normalized to β-actin in TgCRND8 mice cohort (B). (*n* = 5/group; *p* > 0.05, *t* test). **C-D.** Representative immunoblot showing no significant changes in prion protein levels in AAV1- mIL-4 TgCRND8 compared to age-matched controls (C). Intensity analysis of immunoreactive prion protein levels (mean ± sem) was normalized to β-actin (D). (*n* = 5/group; *p* > 0.05, *t* test). **E-F.** Representative anti CT20 immunoblot showing no significant changes in APP and CTFα in 5 month old AAV1-mIL-4 expressing wild type B6/C3H mice compared to age-matched controls (E). Intensity analysis of anti CT20 immunoreactive APP and CTFα levels (mean ± sem) was normalized to β-actin in wild type mice cohort (F). (*n* = 5/group; *p* > 0.05, *t* test).

Analysis of plaque burden showed that there was a 33.5% increase in amyloid plaques in the hippocampus of mIL-4 expressing mice compared to EGFP expressing mice (Figure 2, A-E). There was a concomitant increase in insoluble Aβ levels by biochemical analysis - 41% increase in Aβ42 levels and 55% increase in Aβ40 levels in the SDS extractable Aβ levels (Figure 2, F) respectively and 76% increase in Aβ42 levels and 62% increase in Aβ40 levels in the formic acid extractable Aβ levels respectively (Figure 2, G). Interestingly, the number of Thioflavin S stained “cored” plaques in the hippocampus of mIL-4 expressing mice (15.2% increase) did not increase significantly compared to controls (Figure 3, A-C). We next investigated whether the increase in Aβ was due to changes in APP expression, APP processing, ApoE levels or phagolysosomal dysfunction. Neither APP expression nor CTFα expression or CTFβ levels were altered in mIL-4 expressing TgCRND8 mice compared to controls (Figure 4, A-B). We additionally tested for the levels of endogenous mouse prion protein as the mutant human APP transgene is expressed from mouse prion promoter [22]. No significant change in prion protein levels was apparent between the mIL-4 expressing transgenic mice brains and control cohorts (Figure 4, C-D). Additionally, no change in mouse endogenous APP levels or CTFα levels were seen in 5 month old mIL-4 expressing wild type B6/C3H littermates of TgCRND8 mice injected in the cerebral ventricles on day P2 (Figure 4, E-F), suggesting that mIL-4 does not change either the holo-APP levels or APP processing or prion promoter expression in vivo. Though inflammatory signaling can modulate apoE levels through modulation of Erks [23], no significant change in ApoE levels were seen in mIL-4 injected compared to control transgenic APP mice (Additional file 2: Figure S2, A-B). Autophagic-lysosomal dysfunction may lead to increased accumulation of Aβ [24]. Since anti-inflammatory cytokines, for example, IL-4 and IL-13, have been shown to inhibit autophagy [25], we tested whether changes in autophagic response may account for increased Aβ accrual in mIL-4 expressing mice. No significant changes in the autophagic marker LC3-I were seen in transgenic TgCRND8 mice injected with AAV2/1-mIL-4 (Additional file 3: Figure S3, A, D), though a nonsignificant lowering trend was observed in the nontransgenic cohort (Additional file 3: Figure S3, B, D). We were unable to detect LC3-II in the lysates, making it difficult for us to infer effects of mIL-4 on autophagic flux in vivo. p62 (or sequestosome-1), which is a central player in autophagy was found to be decreased by 47% in mIL-4 expressing mice (Additional file 3: Figure S3, C-D).

A basic tenet of the amyloid cascade hypothesis is that Aβ accumulation triggers the onset and severity of neurodegenerative pathology, including tau hyperphosphorylation. Though occasional CP13 immunoreactive glial cells were visible in both mIL-4 and control mice, mIL-4 mice did not lead to appearance of phosphorylated CP13 immunoreactive tau (phospho Ser202/Thr205) in the hippocampal neurons in TgCRND8 mice, (Additional file 4: Figure S4, A-B). Immunoblotting also failed to show any detectable levels of CP13 or MC6 (phospho Ser235) or PHF1 epitope (phospho Ser396/Ser404) in both cohorts (data not shown).

**Figure 5:**
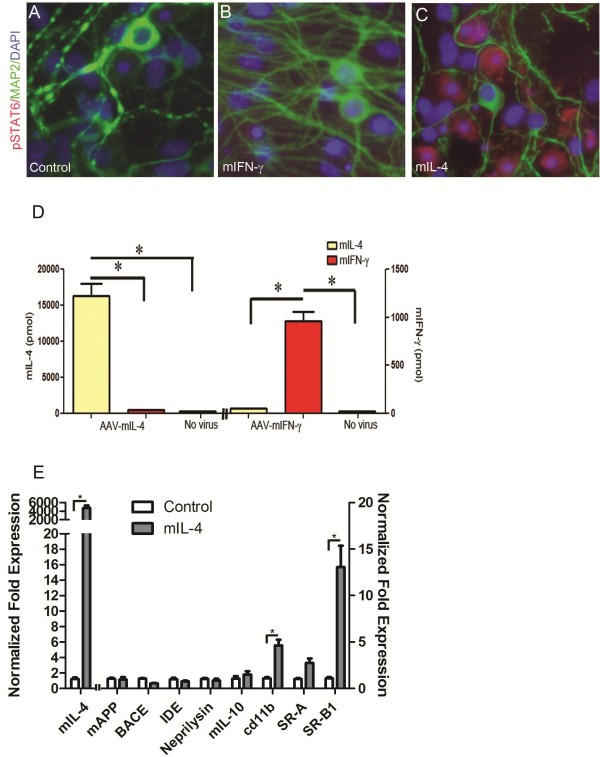
**Characterization of rAAV2/1-mIL-4 treated primary wild type mouse neuroglial culture. A-D.** rAAV2/1-mIL-4 or rAAV2/1-mIFN-γ (2x10^8^ viral genomes) was used to transduce primary mouse neuroglial cultures for 60 hrs in chamber slides. Upregulation of phosphorylated STAT6 (568 nm; red fluorescence) could be seen in mIL-4 expressing cultures (C) but not in mIFN-γ expressing cultures (B) or untreated culture containing no viruses (A). MAP2 (488 nm; green) and DAPI (350 nm, blue) were used to depict neuronal processes and nucleus respectively. mIL-4 and mIFN-γ expression in these cultures was verified by ELISA from media collected from respective cultures (D). Magnification, 400x. (*p < 0.05, *t* test). **E.** Expression of mouse endogenous APP, BACE1, IDE and neprilysin levels are unchanged in rAAV2/1-mIL-4 expressing primary mixed neuroglial cultures compared to untreated control cultures using real time Q-PCR. mIL-4 expression augments scavenger receptors and cd11b levels. Data, expressed as relative levels of mRNA expression, represents averaged fold change values obtained from mIL-4 expressing mice, relative to averaged values obtained from EGFP expressing mice. Data for mIL-4 RNA is plotted on the left x-axis while the rest of the data are plotted on the right x-axis. Data is representative of two independent experiments (*t* test, *p < 0.05). Data represents mean ± sem.

**Figure 6:**
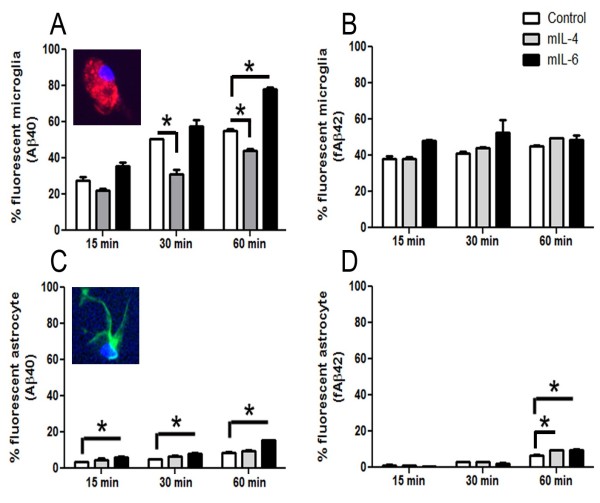
**Differential effect of cytokines on Aβ40 and fAβ42 uptake by primary mouse glia and astrocytes. A-B.** mIL-4 treatment decreases microglial uptake of Aβ40 (A) but does not affect fAβ42 uptake (B). mIL-6 increased microglial Aβ40 uptake (A) but does not significantly alter fAβ42 uptake (B). Primary mouse glia were treated with recombinant cytokines for 10 hrs and incubated with Aβ40-Hilyte555 or Aβ42-Hilyte555 for 15 min, 30 min and 60 min. Trypsinized cells were counted using Accuri6 flow cytometer. Unstained cells and labeled additives were excluded by gating to yield the percentage of fluorescent cells in the mix. Inset depicts cd11b/DAPI stained glia (A) Results are representative of three independent experiments. (*p < 0.05, One way Anova with Tukey’s post test). Data represents mean ± sem. **C-D.** mIL-4 treatment does not affect astrocytic uptake of Aβ40 (C) but increases fAβ42 uptake after 60 min incubation (D). mIL-6 consistently increases astrocytic Aβ40 uptake (C) and only enhances fAβ42 uptake after 60 min incubation (D). Primary mouse astrocytes were treated with recombinant cytokines for 10 hrs and incubated in Aβ40-Hilyte555 or Aβ42-Hilyte555 for 15 min, 30 min and 60 min. Trypsinized cells were counted using Accuri6 flow cytometer. Unstained cells and labeled additives were excluded by gating to yield the percentage of fluorescent cells. Inset depicts GFAP/DAPI labeled astrocyte (C). Results are representative of two independent experiments. (*p < 0.05, One way Anova with Tukey’s post test). Data represents mean ± sem.

To further investigate the mechanism of mIL-4 induced Aβ accumulation, we treated primary wild type mouse neuroglial cultures with rAAV2/1-mIL-4 for 72–80 hours. Analysis of the conditioned media of these cells revealed increased mIL-4 protein (*p* < 0.01, *t* test) but no increases in mIL-6 (data not shown) or mIFN-γ protein compared to control cultures (Figure 5, D). In addition, primary cultures transduced with mIL-4 showed increased levels of nuclear phosphorylated STAT6 compared to mIFN-γ or untreated cultures (Figure 5, A-C). Quantitative RT-PCR analysis confirmed increased mIL-4 (4.5e3 x) levels as well as increased levels of cd11b (6 x), scavenger receptor A (3.3 x) and scavenger receptor B1 (15.6 x) (Figure 5, E). No significant changes in mouse APP, BACE1, or Aβ degrading enzymes (IDE or Neprilysin) were seen (Figure 5, E). Since increased cd11b and scavenger receptors may result in altered phagocytic potential of mIL-4 expressing glia, we performed phagocytosis assays on mouse primary glia and astrocytes treated with medium alone or recombinant mIL-4. Since Aβ40 and Aβ42 can have different effects on phagocytosis, we used both to test out how mIL-4 affects astroglial phagocytois. Primary mouse glia were treated with recombinant cytokines or vehicle for 10 hours, and fresh media added before incubation with fluorescent Aβ for different times (Figure 6, Additional file 5: Figure S5– Additional file 6: Figure S6). These experiments were performed with cultures containing >95% CD45 and Cd11b immunopositive glia (Additional file 5: Figure S5, A-B). Initial microscopic examination showed a decrease in the levels of internalized Aβ40 in mIL-4 treated microglia following 15 min, 30 min or 60 min incubation with Aβ40-555 nm (Additional file 6: Figure S6, A-I). This was confirmed by flow cytometric analysis which showed a significant decrease in internalized fluorescent Aβ40 in mIL-4 treated microglia at these three different timepoints (15 min: -19%; 30 min: -38%; 60 min: -19%) (Figure 6, A; Additional file 5: Figure S5, D-L). Since we have previously shown that mIL-6 treatment augments microglial Aβ phagocytosis [10], we included this as an internal control for Aβ40 internalization in the flow cytometric assay (15 min: +29%; 30 min: +13%; 60 min: +41%) (Figure 6, A; Additional file 5: Figure S5, J- L). Immunoblotting of microglial cells following Aβ40 phagocytosis also showed that mIL-4 treated glia take up Aβ less efficiently (Additional file 5: Figure S5, M-N). In order to test whether fibrillized Aβ42 (fAβ42) has a different effect on glial phagocytosis, we performed phagocytosis assays using Aβ42 fibrils (Figure 6B). Flow analysis shows that neither mIL-6 (15 min: +25.7%; 30 min: +28%; 60 min: +8%) nor mIL-4 (15 min: +0.53%; 30 min: +8.1%; 60 min: +10.4%) significantly affects fAβ42 internalization by microglia (Figure 6B). Since IL-4 can potentially activate astrocytic internalization of Aβ, we performed phagocytosis with murine astrocyte cultures (Figure 6, C-D). Overall, neonatal astrocytes were very inefficient in internalizing Aβ40 as shown earlier [2,26]. Flow cytometric analysis shows that mIL-4 does not significantly alter astrocytic Aβ40 phagocytosis (15 min: +39.5%; 30 min: +50%; 60 min: +12.2%) (Figure 6, C) whereas mIL-6 increases astrocytic Aβ40 internalization significantly at all timepoints tested (15 min: +83.1%; 30 min: +58.3%; 60 min: +85.2%) (Figure 6, C). On the other hand, mIL-4 (15 min: -16.8%; 30 min: +3%) as well as mIL-6 (15 min: -48.4%; 30 min: -26%) has negligible effects on astrocytes phagocytosing fAβ42 at the two earlier timepoints tested (Figure 6, D). Only at the last timepoint tested, i.e., following 60 min of incubation of fAβ42, both mIL-4 (60 min: +44.4%; *p* < 0.05, 1 way Anova) and mIL-6 (60 min: +45%) enhances fAβ42 uptake by primary astrocytes (Figure 6, D).

## Discussion

We have found that overexpression of mIL-4 in the hippocampus of plaque-depositing APP CRND8 mice increased Aβ plaque pathology. We extensively investigated the likely factors that could be responsible for the effects of mIL-4 expression on increased Aβ burden *in vivo*. APP, APP CTF, BACE, ApoE and Aβ degrading enzyme levels did not appear to be altered. As IL-4 has been reported to inhibit autophagy [27], we investigated LC3I/II levels, but this was not informative, as we could not reliably detect LC3II. Levels of another key regulator of autophagy, p62, were decreased by mIL-4. As p62 has pleiotropic functions in addition to its role in autophagy [28] the significance of this finding is unclear. Furthermore if autophagic pathways were being inhibited, p62 would be expected to increase, not decrease. Given i) that these data indicating that mIL-4 does not appear to be affecting APP processing and is not altering major Aβ chaperones or degrading enzymes and ii) that we and others have previously linked proinflammatory activation of microglia to reduced plaque burdens and enhanced microglial scavenging of Aβ [10,11,13,14,17,29,30], we hypothesize that mIL-4 increases Aβ burden via reductions in glial scavenging of Aβ. We explored this hypothesis through Aβ internalization studies in primary microglia and astrocytes cultures. These studies do show that mIL-4 abrogates soluble Aβ40 uptake by microglia without affecting aggregated fAβ42 phagocytosis. Additionally, mIL-4 does not affect the uptake of soluble or aggregated Aβ by astrocytes, though on longer incubation, there was an increasing trend in Aβ ingestion by the astrocytes. Given the low number of astrocytes internalizing Aβ and the fact that astrocyte cultures may contain ~10-20% microglia, it is difficult to conclude whether astrocytes by themselves have a critical role in Aβ phagocytosis.

Microglia can scavenge Aβ via receptor-mediated phagocytosis as well as macropinocytosis [26]. In vivo imaging techniques have demonstrated that microglia can home onto newly appearing plaques and internalize Aβ [31]. Microglial interaction with Aβ occurs through several cell surface receptors, for example, SR-A, SR-B, integrins and Toll-like receptors [15,32-34]. Previous studies have noted that inflammatory cytokines decrease scavenger receptors levels in microglial cell lines and inhibit Aβ uptake [35]. We find that mIL-4 expression leads to increased SRs in mixed neuroglial cultures, but this appears to be linked to modest inhibitory effects on soluble, but not aggregated, Aβ uptake by microglia. Therefore, it is possible that in the absence of inflammatory mediators, these cells are not competent for Aβ internalization and degradation, even if they express the receptors [36,37]. Indeed, microglia were found to require stimulation with cytokines or opsonins for internalizing and degrading Aβ efficiently in amyloid vaccination paradigms [38]. Microglial populations from human AD brains display an IL-4/IL-13 induced alternate activation profile [39] as well as increased IL-1β, suggesting that an imbalance between the inflammatory “classical” and anti-inflammatory alternate activation profiles may result in a dysfunctional glial response and “failed” phagocytosis. Although speculative, these studies may have relevance to human therapy. Many studies show an association of nonsteroidal anti-inflammatory drugs (NSAIDS) use with reduction of dementia risk [40]. However, in the Adult Changes in Thought (ACT) study, there was an increased incidence of all-cause dementia and AD dementia in individuals with heavy nonselective NSAID use, and this was associated with an increase in neuritic plaques at autopsy [41]. Our data could provide an explanation for this association.

It is generally accepted that an altered innate immune response is an inherent feature of AD and most, if not all, degenerative, CNS proteinopathies [42]. Though a large body of literature on this subject refers to this altered immune response as “pathogenic” or “harmful”, in many cases this relationship is assumed and has not been formally proven. Increased pro-inflammatory cytokines levels (TNFα, Interleukin-6, Interleukin-1β), combined with decreased levels of anti-inflammatory cytokines (Interleukin-10), have been correlated with cognitive deficits suggesting that early inflammatory changes may be detrimental [43]*.* Furthermore proinflammatory stimuli have been linked to overt neuronal degeneration [44]. Although proinflammatory factors may have a detrimental role on neuronal function, priming of CNS microglia and possibly astrocytes by accumulating Aβ, may be an essential innate host response that triggers glial phagocytosis activity and clears Aβ deposits. Indeed, we and others have previously shown that activation of the innate immune signaling pathways can attenuate Aβ accumulation in APP transgenic mice and alter the disease process [10-14,17,29,30].

mIL-4 has been shown to enhance Aβ phagocytosis in primary rodent glial cultures in vitro [45,46]. Based largely on such evidence, promotion of a glial M2 phenotype has been proposed to be beneficial in preclinical AD models and this concept was directly supported by one recent study, using a similar AAV mediated IL-4 expression paradigm as used in this present study [4]. In this study IL-4 induced a Th2 like glial phenotype, but in contrast to our results, Kiyota et al, showed that IL-4 expression in the hippocampus led to mitigation of Aβ pathology and improved behavior in APP/PS1 mice [4]. Inherent differences in transgenic mice as well as experimental paradigms may underlie the disparate observations. It is possible that presenilins may regulate cytokine secretion [47], and mutant PS1 especially may confer altered sensitivity to immune challenges in the resident glial cells [48]. Additionally, the two studies were distinct with respect to initiation of treatment. Kiyota and colleagues performed a primary prevention study in which IL-4 expression was initiated in the pre-deposition phase, whereas IL-4 expression in our studies was initiated in mice with modest levels pre-existing plaques. In any case, additional studies of both IL-4 and other anti-inflammatory factors will be required to address this discrepancy and the generalizability of this finding.

## Conclusion

In summary, we demonstrate that focal overexpression of mIL-4 in APP transgenic mouse brains leads to exacerbated Aβ plaque pathology. As these results are opposite of effects observed with proinflammatory cytokines, we would suggest that the underlying mechanism appears to be at least in part a failure of glia to successfully clear Aβ. Our data points to the complex relationship between microglial phenotype and the final functional outcome, necessitating a more cautious and thorough examination of potential anti-inflammatory therapies for AD.

## Methods

*Mice.* All animal husbandry procedures performed were approved by the Institutional Animal Care and Use Committee.

*AAV1 preparation and injection*. AAV1 viruses expressing mIL-4 or EGFP, under the control of the cytomegalovirus enhancer/chicken β actin promoter were generated as described previously [19]. For stereotaxic injections, TgCRND8 mice (n = 6/group) were anesthetized with 1.5% isoflurane in 1% oxygen and secured into a Kopf apparatus (Model 900 Small Animal Stereotaxic Instrument*,* David Kopf Instruments). The coordinates for injection were −1.7 caudal, -1.6 lateral and −1.2 ventral from the bregma. A UMP2 Microsyringe Injector and Micro4 Controller (World Precision Instruments, USA) was used to inject 2 μl (10^10^ viral genomes) of virus at a constant rate over a 10 minute period. After allowing an additional 10 minutes, the needle was raised and the scalp incision was closed aseptically. Nontransgenic mice used in the study were injected with rAAV2/1-mIL-4 on day P2 into the cerebral ventricles and aged till 5 months of age.

*Quantitative real-time PCR.* Total RNA from mice hippocampus or primary wild type mouse neuroglial cultures was isolated using the RNaqueous kit (Ambion) and reverse transcribed using Superscript III (Invitrogen). The Q-PCR (initial denaturation cycle of 95 °C /10 min, followed by 40 amplification cycles of 95 °C /15 s and 60 °C/ 1 min) was performed with ABI Prism 7900 Real Time PCR System (Applied Biosystems) using SYBR Green to detect the amplification products. Relative quantification of mRNA expression was calculated by the ΔC_T_ method described by the manufacturer (ABI Prism 7700 Sequence Detection System, User Bulletin #2) after adjusting the levels to the corresponding internal actin control for each sample. Primers and probes were designed following Roche Universal Probe Library sequences (Hoffmann-La Roche, Germany).

*Preparation of brain homogenate for immunoblotting and Aβ ELISA assay.* Brain were coronally dissected 1 mm anterior and posterior to the point of injection and used for subsequent analysis. Thus, the samples for immunoblotting were obtained by dissecting the hippocampus and overlying cortex and thalamus of injected brains. Protein samples (RIPA soluble or 2% SDS soluble) separated on Bis-Tris 12% XT gels (Bio-Rad, USA) were probed with the antibody CT20 (anti-APP C-terminal 20 amino acid; T. E. Golde; 1:1000); 82E1 (IBL, 1:500), Prion (Abcam; 1:1000); LC3 (MBL; 1:500; Cell Signaling, 1:250; Novus, 1:500); p62 (Cell Signaling, 1:250), CP13 (P. Davies; 1:500) and anti β-actin (Sigma,1:1000). Relative band intensity was quantified using ImageJ software (NIH).

Aβ levels were determined biochemically using human Aβ end-specific sandwich ELISA as previously described [19] on samples sequentially extracted with RIPA buffer, 2% SDS and 70% Formic Acid. All ELISA results were analyzed using SoftMax Pro software (Molecular Device).

*Immunohistochemical imaging and image processing.* Brains were coronally dissected at the point of injection for analysis. Immunohistochemical staining was done using pan Aβ antibody 33.1.1 (1:1500, T. Golde), 82E1 (1:500, IBL), Iba-1 (1:1000; Wako), GFAP (1:500; Chemicon), pSTAT6 (1:500, Cell Signaling) and MAP2 (1:1000; Chemicon). 1% Thioflavin S (Sigma) staining was done on paraffin embedded brain sections using established protocols.

Immunohistochemically and fluorescent stained sections were captured using the Aperio Scanscope XT or FL image scanner and analyzed using either Aperio positive pixel count or ImageJ program. Brightness and contrast alterations were applied identically on captured images using Adobe Photoshop CS3.

*Quantification of Aβ deposition and gliosis.* Paraformaldehyde fixed paraffin embedded brain tissue sections were immunostained with 33.1.1 antibody. Aβ plaque burden and intensity of astrogliosis staining was calculated using the Positive Pixel Count program (Aperio). At least three sections per sample, 30 μm apart, were averaged by a blinded observer to calculate plaque burden. For Thioflavin S quantitation, one section per sample was used by a blinded observer to manually count the plaques using Adobe Photoshop CS5.

*Primary murine culture and microglia/astrocyte phagocytosis assay.* Primary microglia, astrocytes or neuronal cultures (mixed with astrocytes) were obtained from cerebral cortices of wild type neonate mice as described previously [38]. Glial and astrocytic cultures typically were >90% cd11b (cd11b-APC; 1:200, BD Biosciences) or GFAP (Sigma, 1:500) immunopositive respectively. For phagocytosis assays, microglia or astrocytes, pre-treated with mIL-4 (R&D Systems, USA; 5 ng/ml for 10 hours) or mIL-6 (R&D Systems, USA; 10 ng/ml for 10 hours) were incubated with 0.5 μM Hilyte555-Aβ40 (Anaspec, USA) or fibrillar Hilyte555-Aβ42 (Anaspec, USA). Recombinant fluorescent Aβ was resuspended in DMSO to 1 mg/ml and diluted in DMEM medium to 0.5 μM before addition to cells. Hilyte555-Aβ42 was fibrillized at 37^0^ C for 6 hours in PBS. Cells were analyzed at three timepoints following addition of Aβ to the culture at 37^0^ C: 15 min, 30 min and 1 hr. Cells were washed, gently trypsinised to remove cell surface associated fluorescent entities, fixed in paraformaldehyde and mounted in DAPI containing medium for visualization. Fluorescence intensity of individual cells from at least 5 or more fields of views per sample were calculated from Image J and averaged. For FACS analysis, following washes, cells were collected by trypsinization and resuspended in FACS buffer containing BSA and scanned. Scans were collected using Accuri6 and analyzed with FCS Express 4 Flow Research (BD Biosciences). Neuronal cultures were transduced with AAV (mIL-4 or mIFN-γ; 10^8^ viral genomes) on day 7 for 60 hours and fixed with paraformaldehyde for subsequent immunocytometry. Cytokine ELISA was performed using BD Bioscience OptiEIA reagents.

### Statistical analysis

One-way Anova (with Tukey’s post-hoc test) or two-tailed Student's t test was used for statistical comparison (SigmaStat 3.0 version). Graphical analyses were done using Prism 4 (GraphPad Software) and final images created using Photoshop CS2 (Adobe).

## Abbreviations

AAV, Adeno-associated virus; ACT, Adult Changes in Thought; AD, Alzheimer’s disease; Aβ, Amyloid β; APP, Amyloid β precursor protein; ApoE, Apolipoprotein ϵ4; BACE, β-site APP cleaving enzyme; CTF, C-terminal fragment; EGFP, Enhanced green fluorescent protein; Erk, Extracellular signal-regulated kinases; FACS, Fluorescence activated cell sorting; GFAP, Glial fibrillary acidic protein; IL-4, Interleukin-4; IFN-γ, Interferon-γ; IACUC, Institutional animal care and use committee; Iba-1, Ionized calcium binding adaptor protein 1; LC3, Microtubule-associated protein light chain 3; NSAIDS, Nonsteroidal anti-inflammatory drugs; SR, Scavenger receptor; STAT, Signal Transducer and Activator of Transcription; TNFα, Tumor necrosis factor α.

## Competing interests

The authors declare no competing interests.

## Authors’ contributions

PC conducted the experiments and wrote the manuscript; LT performed immunostaining, primary mouse neuroglial culture, phagocytosis assays and flow cytometry; AB performed mouse brain Q-PCR and amyloid burden analysis; CC-D prepared recombinant AAV and Q-PCR of neuroglial culture; PD provided helpful discussion; TEG coordinated the research, supervised the project and assisted in manuscript preparation. All authors have read and approved the final manuscript.

## Supplementary Material

Additional file 1 **Figure S1.** rAAV2/1-EGFP expression in TgCRND8 mice hippocampus. **A-I.** Representative image obtained from mice stereotactically injected with AAV2/1-EGFP in the hippocampus. 4 month old TgCRND8 were injected into the hippocampus and analyzed after 6 weeks. Representative images of EGFP immunoreactivity on paraffin embedded whole brain section (A), cortex (B), hippocampal CA neurons (C-E), midbrain, (F), cerebellum (G), thalamus (H) and olfactory bulb (I) are shown. Representative hippocampus from uninjected mice is shown as control (A, inset). I-V, cortex layers I to V; CC, corpus callosum; Sp, Septum; Th, thalamus. *Scale Bar,* 600 μm (A) and 85 μm (B-I). Click here for file

Additional file 2 **Figure S2.** mIL-4 expression does not alter ApoE levels. **A-B.** No significant change in ApoE levels was seen in the hippocampus of mIL-4 expressing 5.5 month old transgenic CRND8 mice or age-matched control cohorts (A). Intensity analysis of ApoE levels was normalized to β-actin (B). (*n* = 5-6/group; *t* test, *p* > 0.05). Click here for file

Additional file 3 **Figure S3.** mIL-4 expression does not alter levels of the autophagic marker LC3 but decreases p62 protein levels.** A-B.** No significant change in the autophagic marker (microtubule-associated protein light chain 3, LC3) was seen in mIL-4 expressing 5.5 month old transgenic CRND8 mice (TG, A) or nontransgenic 5 month old nontransgenic cohorts (NTG, B). **C.** p62 protein levels decrease in mIL-4 expressing 5.5 month old transgenic CRND8 mice (TG). **D.** Intensity analysis of anti LC3 and p62 immunoreactive band after normalization to β-actin immunoreactivity. (*n* = 4-6/group). (*p < 0.05; *t* test). Click here for file

Additional file 4 **Figure S4.** mIL-4 expression does not alter levels of phosphorylated tau. No significant change in phosphorylated tau (using CP13 antibody) was seen in the hippocampus of mIL-4 expressing 5.5 month old transgenic CRND8 mice (**B**) or age-matched control cohorts (**A**). Occasional CP13 immunoreactivity was seen in glial cells in the pyramidal layer of the hippocampus (inset). *Scale Bar,* 150 μm (A, B) and 25 μm (inset). (*n* = 6/group). Click here for file

Additional file 5 **Figure S5.** Analysis of primary mouse microglial culture following Aβ40 phagocytosis.** A-B.** Representative flow cytometric analysis of unstained (A) and CD45-FITC stained mouse microglia (B). Glial populations used for subsequent phagocytosis experiments were >95% positive for both CD45 and cd11b. Inset in A depicts a representative unstained primary mouse glial culture. Magnification, 200x. **C-L.** Primary mouse glia were stimulated with medium alone (Control; D-F) or recombinant mIL-4 (5 ng/ml; G-I) or mIL-6 (10 ng/ml; J-L). After 10 hrs of stimulation, cells were incubated with fluorescent Aβ40 for 15 min (D, G, J), 30 min (E, H, K) or 60 min (F, I, L). Cells were trypsinized for the analysis of internalized fluorescent Aβ40 (D-L) by FACS. Unstained cells and additives have been gated for exclusion (C). Representative data from three independent experiments have been shown. Quantified data has been plotted in Figure 6A. **M-N.** Immunoblot analysis to detect presence of Aβ in primary glial cells at different timepoints following Aβ40 phagocytosis in the presence or absence of mIL-4 (M). Intensity analysis of 82E1 immunoreactive Aβ monomer after normalization to β-actin immunoreactivity has been shown (N). Representative data from two independent experiments have been shown. Click here for file

Additional file 6 **Figure S6.** Microscopic analysis of Aβ40 phagocytosis by mIL-4 treated mouse primary glia. Primary mouse glia were treated with 5 ng/ml mIL-4 for 10 hrs and incubated with fluorescent Aβ40 for 0 min (A-B), 15 min (C-D), 30 min (E-F) or 60 min (G-H). Following gentle trypsinization, cells were fixed, stained with the nuclear stain DAPI and visualized. Inset (C) depicts mIL-6 treated glia after 15 min incubation with Aβ40. Quantitation of average fluorescent count is depicted (I). Magnification, 400x. (*p < 0.05; *t* test). Click here for file
