# Supplementary figures and images for: Hippocampal expression of murine IL-4 results in exacerbation of amyloid deposition
Source: Mol Neurodegener. 2012 Jul 29;7:36. doi: 10.1186/1750-1326-7-36 (PMC3441281; doi:10.1186/1750-1326-7-36)

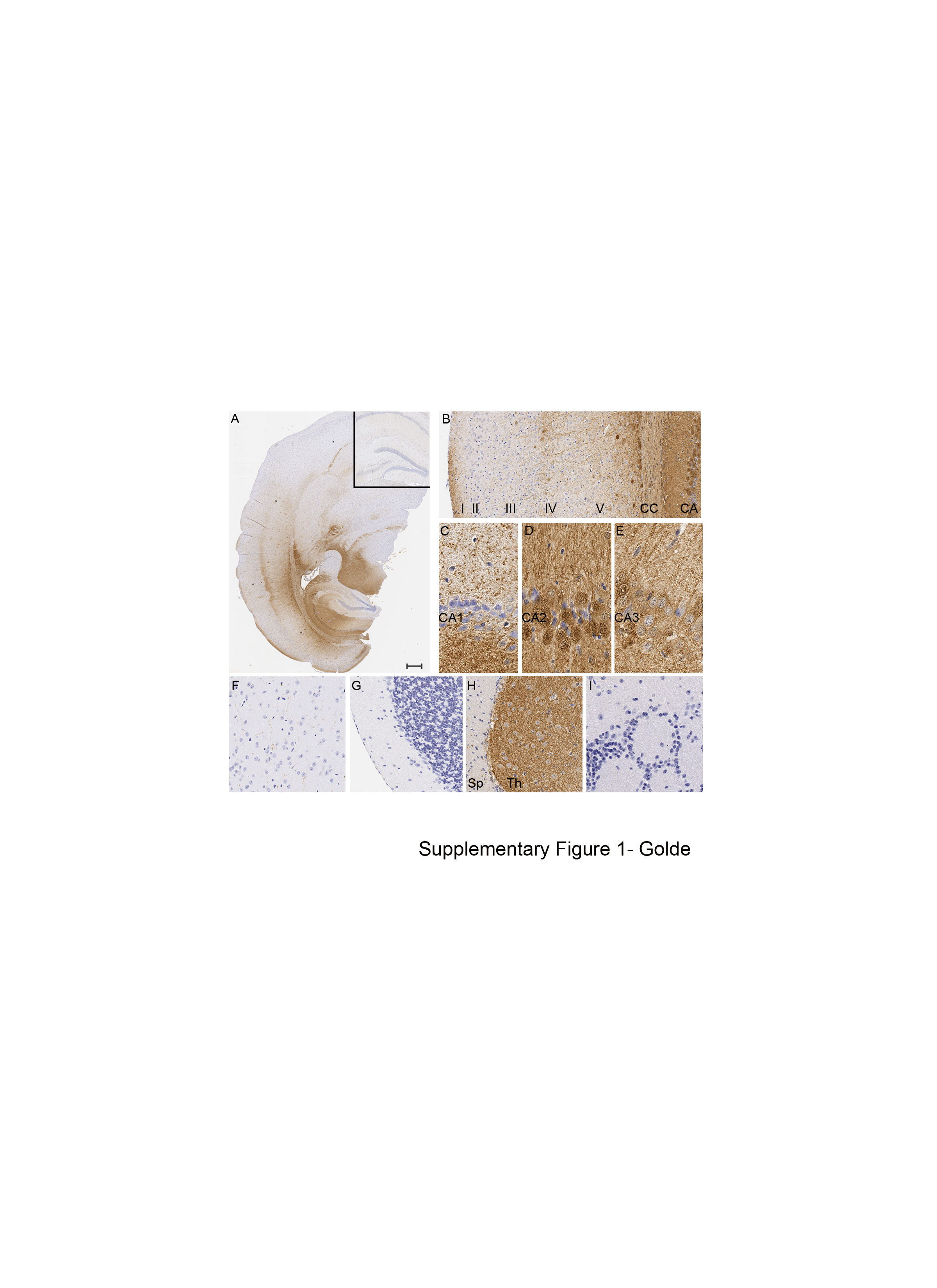

Supplement: Additional file 1 — Figure S1. rAAV2/1-EGFP expression in TgCRND8 mice hippocampus. A-I. Representative image obtained from mice stereotactically injected with AAV2/1-EGFP in the hippocampus. 4 month old TgCRND8 were injected into the hippocampus and analyzed after 6 weeks. Representative images of EGFP immunoreactivity on paraffin embedded whole brain section (A), cortex (B), hippocampal CA neurons (C-E), midbrain, (F), cerebellum (G), thalamus (H) and olfactory bulb (I) are shown. Representative hippocampus from uninjected mice is shown as control (A, inset). I-V, cortex layers I to V; CC, corpus callosum; Sp, Septum; Th, thalamus. Scale Bar, 600 μm (A) and 85 μm (B-I). [file 1750-1326-7-36-S1.jpeg]

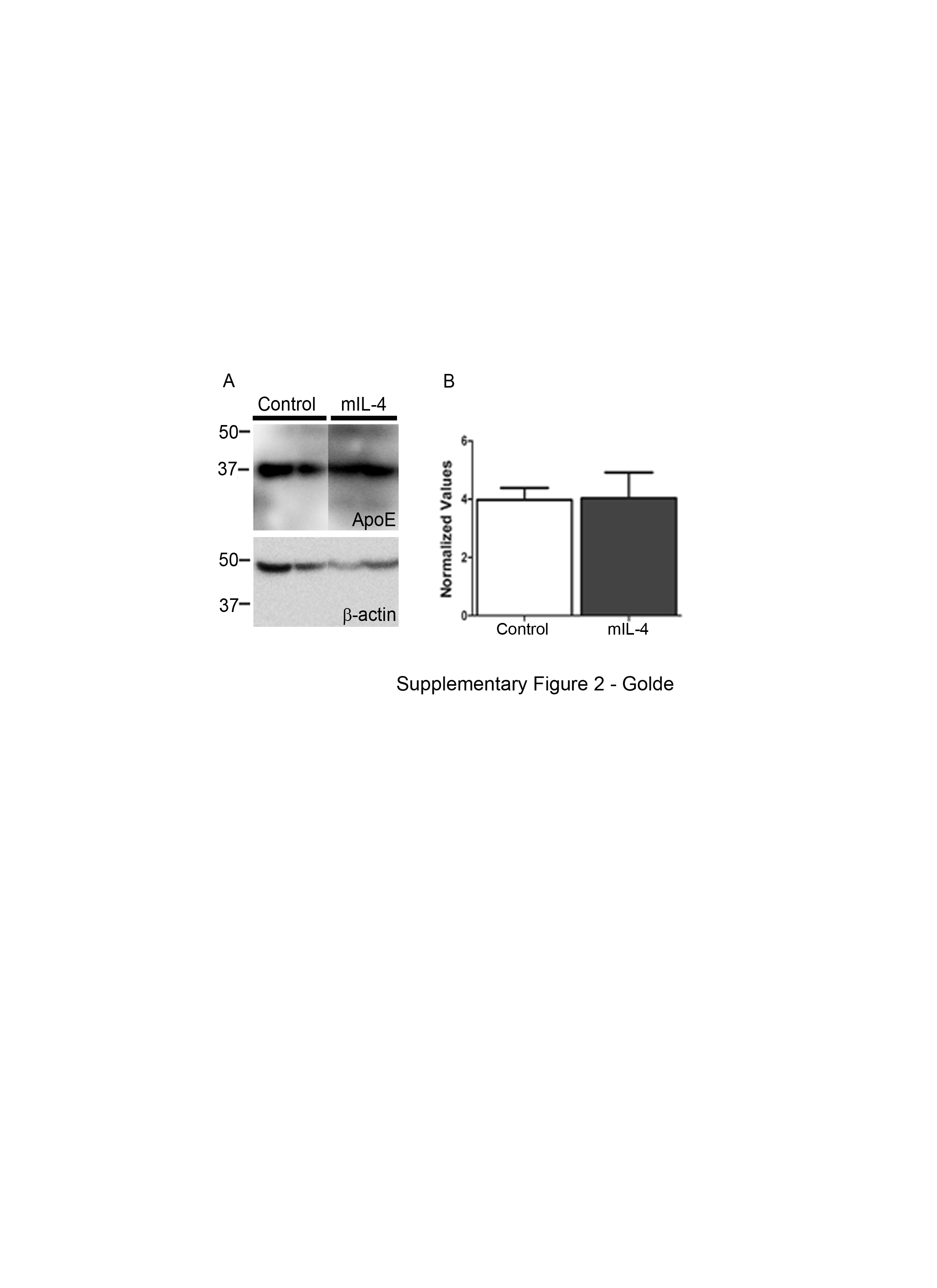

Supplement: Additional file 2 — Figure S2. mIL-4 expression does not alter ApoE levels. A-B. No significant change in ApoE levels was seen in the hippocampus of mIL-4 expressing 5.5 month old transgenic CRND8 mice or age-matched control cohorts (A). Intensity analysis of ApoE levels was normalized to β-actin (B). (n = 5-6/group; t test, p > 0.05). [file 1750-1326-7-36-S2.tiff]

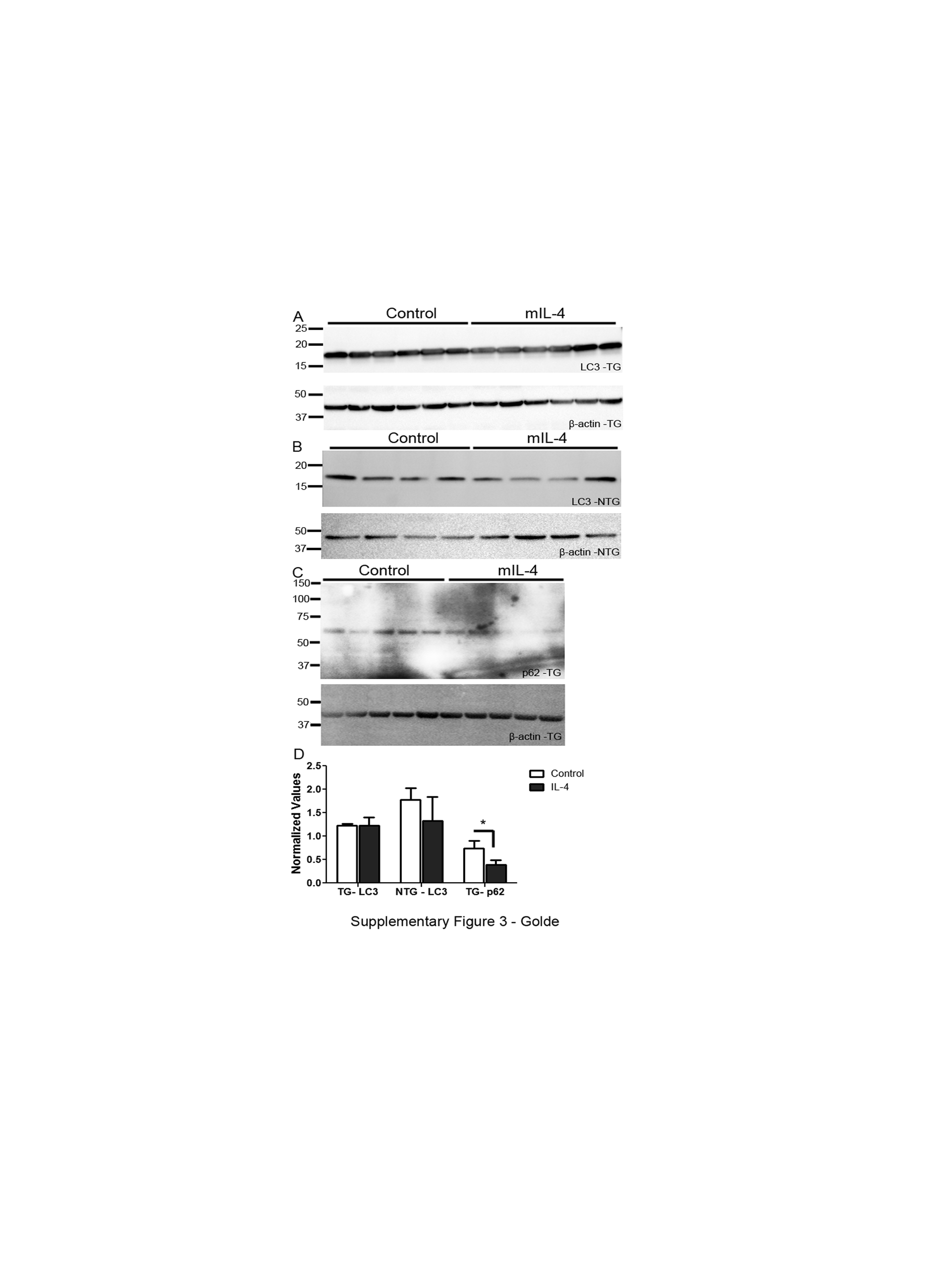

Supplement: Additional file 3 — Figure S3. mIL-4 expression does not alter levels of the autophagic marker LC3 but decreases p62 protein levels. A-B. No significant change in the autophagic marker (microtubule-associated protein light chain 3, LC3) was seen in mIL-4 expressing 5.5 month old transgenic CRND8 mice (TG, A) or nontransgenic 5 month old nontransgenic cohorts (NTG, B). C. p62 protein levels decrease in mIL-4 expressing 5.5 month old transgenic CRND8 mice (TG). D. Intensity analysis of anti LC3 and p62 immunoreactive band after normalization to β-actin immunoreactivity. (n = 4-6/group). (*p < 0.05; t test). [file 1750-1326-7-36-S3.tiff]

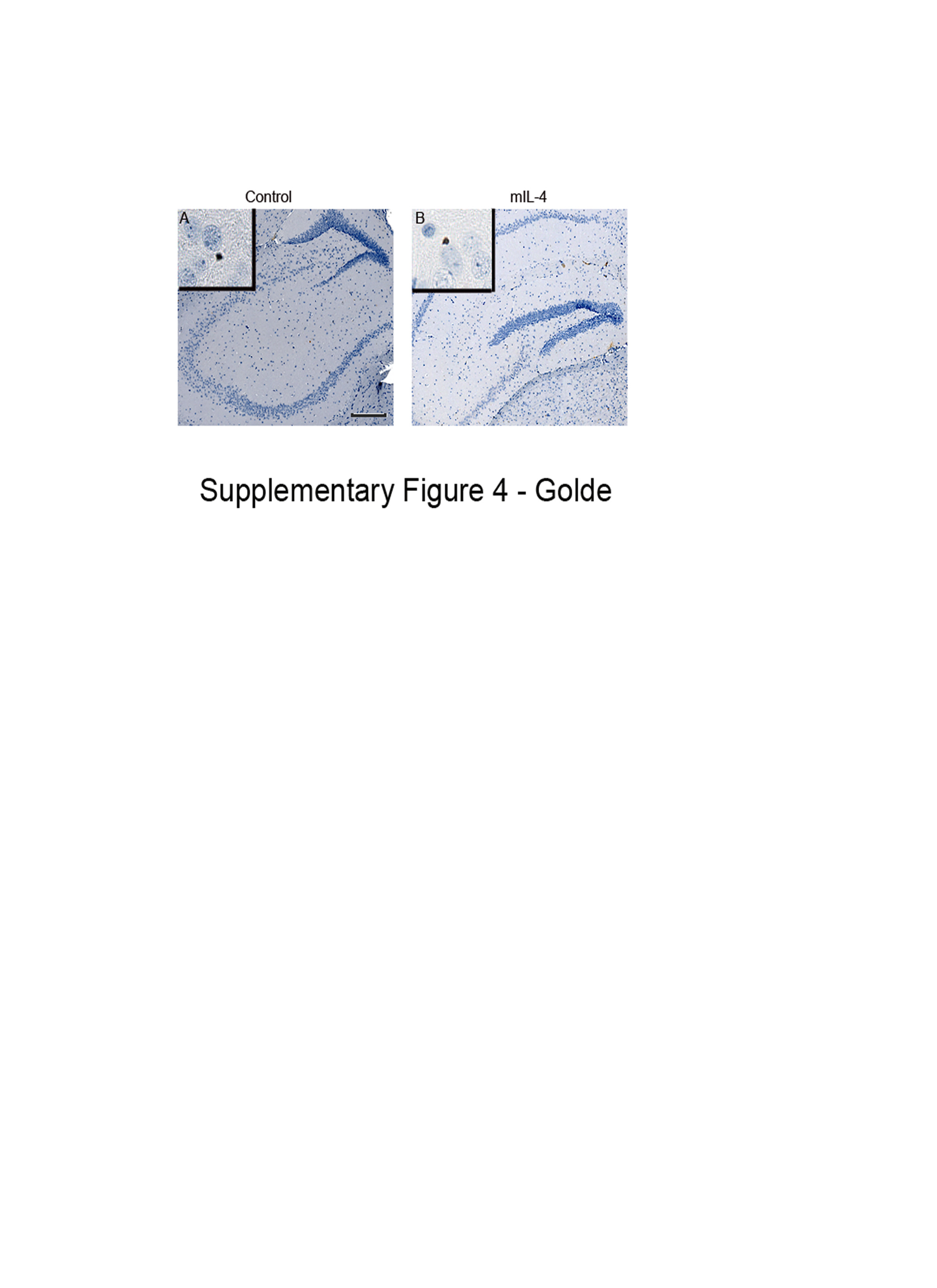

Supplement: Additional file 4 — Figure S4. mIL-4 expression does not alter levels of phosphorylated tau. No significant change in phosphorylated tau (using CP13 antibody) was seen in the hippocampus of mIL-4 expressing 5.5 month old transgenic CRND8 mice (B) or age-matched control cohorts (A). Occasional CP13 immunoreactivity was seen in glial cells in the pyramidal layer of the hippocampus (inset). Scale Bar, 150 μm (A, B) and 25 μm (inset). (n = 6/group). [file 1750-1326-7-36-S4.jpeg]

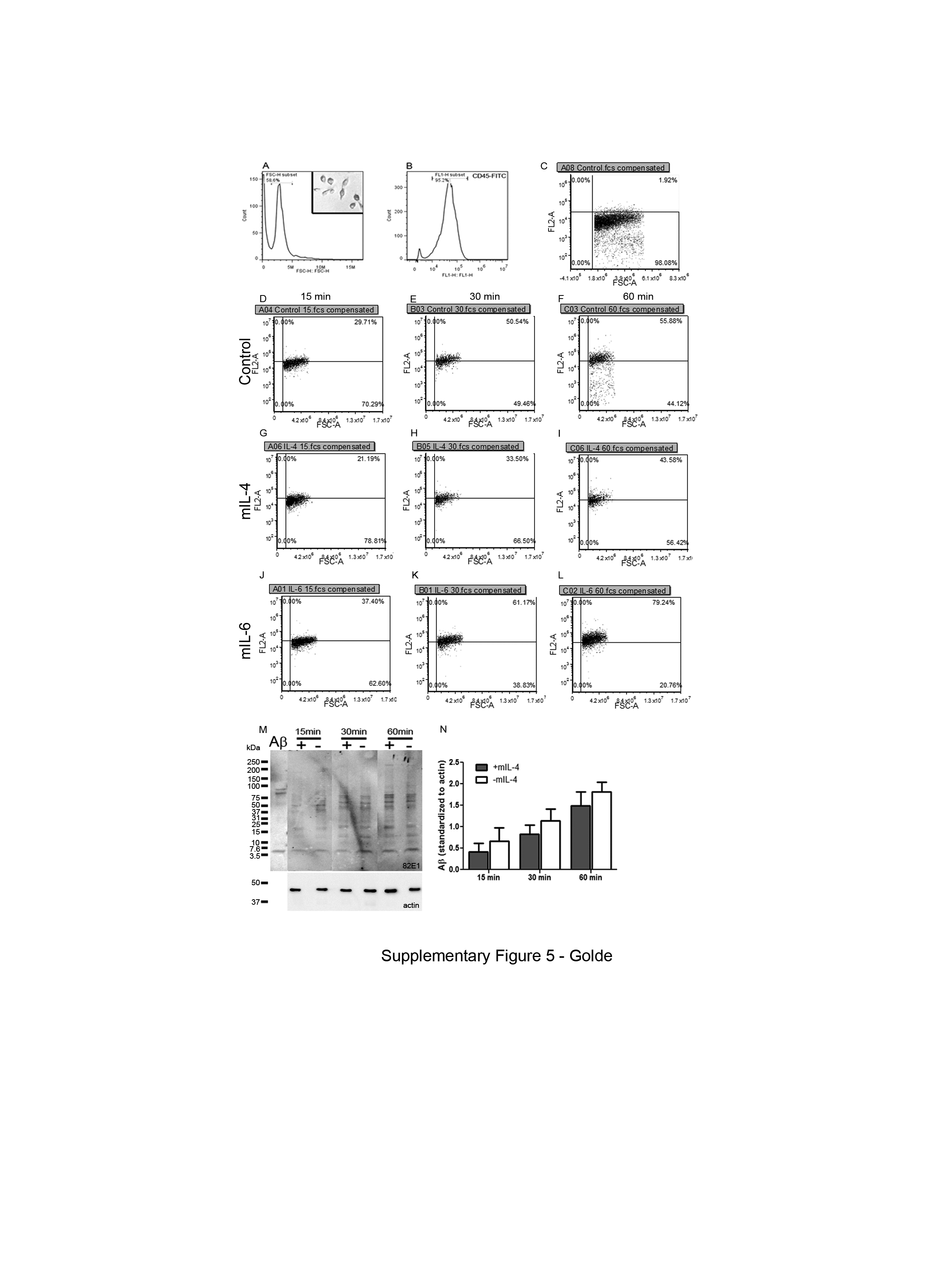

Supplement: Additional file 5 — Figure S5. Analysis of primary mouse microglial culture following Aβ40 phagocytosis. A-B. Representative flow cytometric analysis of unstained (A) and CD45-FITC stained mouse microglia (B). Glial populations used for subsequent phagocytosis experiments were >95% positive for both CD45 and cd11b. Inset in A depicts a representative unstained primary mouse glial culture. Magnification, 200x. C-L. Primary mouse glia were stimulated with medium alone (Control; D-F) or recombinant mIL-4 (5 ng/ml; G-I) or mIL-6 (10 ng/ml; J-L). After 10 hrs of stimulation, cells were incubated with fluorescent Aβ40 for 15 min (D, G, J), 30 min (E, H, K) or 60 min (F, I, L). Cells were trypsinized for the analysis of internalized fluorescent Aβ40 (D-L) by FACS. Unstained cells and additives have been gated for exclusion (C). Representative data from three independent experiments have been shown. Quantified data has been plotted in Figure 6A. M-N. Immunoblot analysis to detect presence of Aβ in primary glial cells at different timepoints following Aβ40 phagocytosis in the presence or absence of mIL-4 (M). Intensity analysis of 82E1 immunoreactive Aβ monomer after normalization to β-actin immunoreactivity has been shown (N). Representative data from two independent experiments have been shown. [file 1750-1326-7-36-S5.tiff]

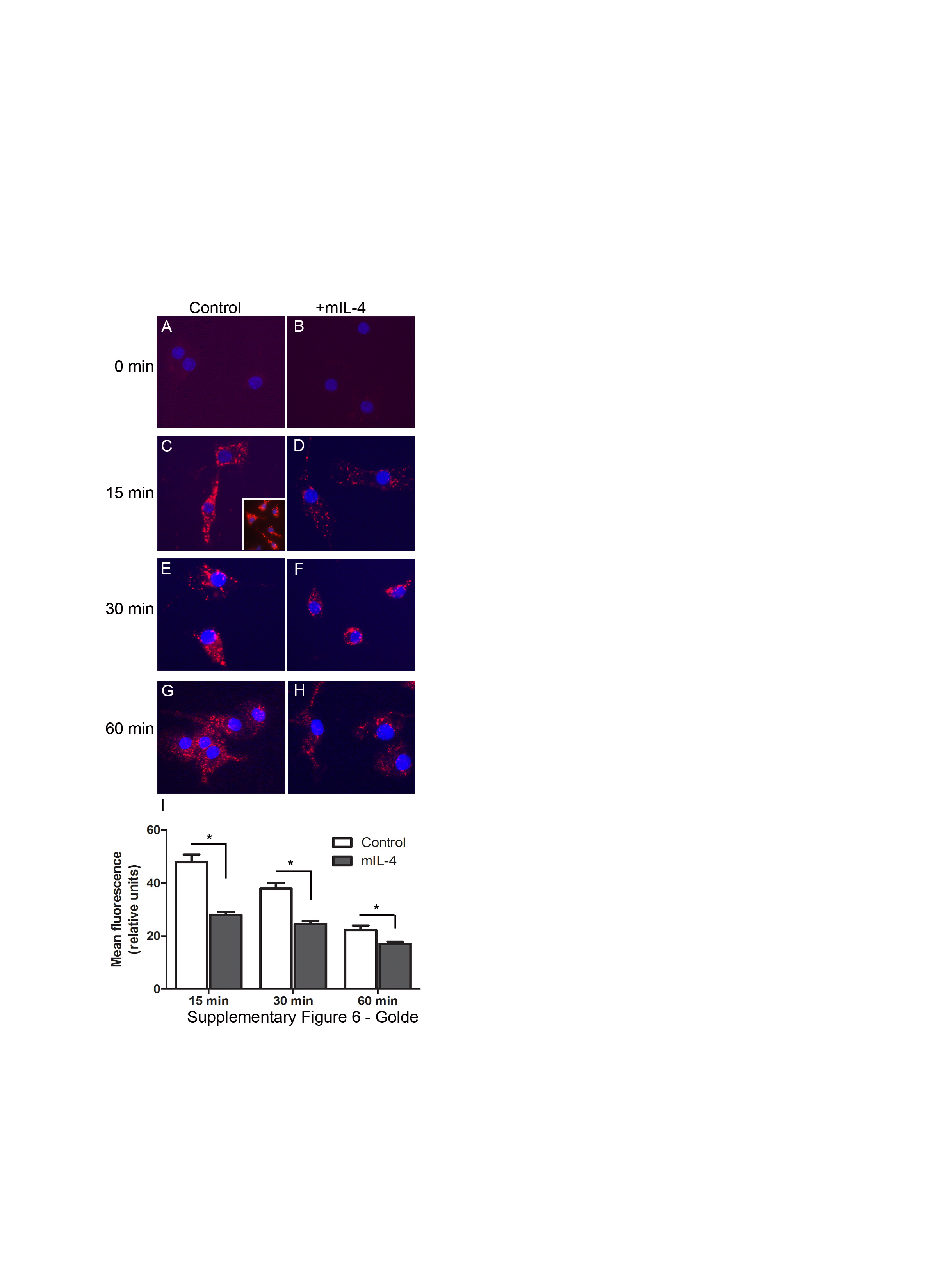

Supplement: Additional file 6 — Figure S6. Microscopic analysis of Aβ40 phagocytosis by mIL-4 treated mouse primary glia. Primary mouse glia were treated with 5 ng/ml mIL-4 for 10 hrs and incubated with fluorescent Aβ40 for 0 min (A-B), 15 min (C-D), 30 min (E-F) or 60 min (G-H). Following gentle trypsinization, cells were fixed, stained with the nuclear stain DAPI and visualized. Inset (C) depicts mIL-6 treated glia after 15 min incubation with Aβ40. Quantitation of average fluorescent count is depicted (I). Magnification, 400x. (*p < 0.05; t test). [file 1750-1326-7-36-S6.jpeg]
